# Assessment of prison life of persons with disability in Ghana

**DOI:** 10.1186/s12914-016-0094-y

**Published:** 2016-08-08

**Authors:** Joslin Dogbe, Ellis Owusu-Dabo, Anthony Edusei, Gyikua Plange-Rhule, Nicholas Addofoh, Sandra Baffour-Awuah, Osei Sarfo-Kantanka, Charles Hammond, Michael Owusu

**Affiliations:** 1 Centre for Disability and Rehabilitation Studies, Department of Community Health, Kwame Nkrumah University of Science and Technology, Kumasi, Ghana; 2 Kumasi Centre for Collaborative Research in Tropical Medicine, Kumasi, Ghana; 3 Department of Child Health, Komfo Anokye Teaching Hospital, P. O. Box 1934, Kumasi, Ghana; 4 Department of Internal Medicine, Komfo Anokye Teaching Hospital, Kumasi, Ghana; 5 Department of Clinical Microbiology, Komfo Anokye Teaching Hospital, Kumasi, Ghana

**Keywords:** Disability, Ghana, Prisons

## Abstract

**Background:**

Persons with Disabilities (PWDs) are a unique group that are often overlooked in many developing countries due to systemic weaknesses, lack of political commitment and inadequate support from government and non-governmental agencies. The population of these individuals is however steadily on the increase and currently corresponds to 15 % of the world population. Although much data exist on lifestyle and conditions of prisoners with disabilities in the western world, scanty information is available in Africa. In Ghana, there is insufficient data on the occurrence and social characteristics of prisoners with disabilities. The purpose of this current study was therefore to identify the occurrence, types and causes of disabilities among prisoners serving sentences in Ghanaian prisons.

**Methods:**

This study was a descriptive cross-sectional survey conducted in the Male and Female Regional Prisons in Kumasi, Sunyani and the Nsawam Medium Security Prison, from November to December 2011. PWDs were selected by prisons officers and interviewed using structured questionnaires on variables such as socio-demographic characteristics, causes of disabilities and accessibility to recreational facilities. Ethical approval was obtained from the security services and the Committee of Human Research Publications and Ethics (CHRPE) of the School of Medical Sciences, Kwame Nkrumah University of Science and Technology (KNUST).

**Results:**

We screened 6114 records of prisoners of which 1852 (30.3 %) were from the Kumasi Central Prisons, 3483 (57 %) from the Nsawam Medium Security and 779 (12.8 %) from the Sunyani Central Prisons. A total of 99 PWDs were identified with the commonest disability being physical, followed by visual, hearing, speech, mental and albinism. Most of the disabilities were caused by trauma (68.8 %) followed by infection (16.7 %), and drug related mental disabilities (6.3 %). Fifty (50.5 %) out of the 99 PWDs were not provided with assistive devices although they admitted the need for such.

**Conclusion:**

The present study has demonstrated the occurrence and conditions of PWDs in Ghanaian prisons. Major stakeholders including government agencies and other organisations could develop policies that would improve the conditions and livelihood of prisoners with disabilities in Ghana.

## Background

The *International Classification of Functioning, Disability and Health* (ICF) defines disability as an umbrella term for impairments, activity limitations, and participation restrictions. Specifically it refers to the negative aspects of the interaction between individuals with a health condition and the internal and external environmental factors [1].

There are over one billion Persons with Disabilities (PWDs) in the world, of whom between 110 and 190 million experience very significant difficulties. This corresponds to about 15 % of the world’s population and is higher than previous World Health Organization (WHO) estimates [2].

An estimated 40 % of the African population are believed to consist of people with disabilities with 15 % of these being school-age children [3, 4].

The prevalence of disability is growing due to population ageing and the global increase in chronic health conditions. Patterns of disability in a particular country are influenced by trends in health conditions and trends in environmental and other factors, such as road traffic crashes, natural disasters, conflict, diet and substance abuse [2].

PWDs include those who have long-term physical, mental, intellectual or sensory impairments that affect their daily routine activities. These individuals are among the most vulnerable individuals in our society yet they are visible in all levels of the community. Some of them are street people and subsist in extreme poverty thus depriving them of rehabilitation devices. Data from some Southern African countries found that only 26–55 % of people received the medical rehabilitation they needed; 17–37 % received the assistive devices they needed; 5–23 % received the vocational training they needed; and 5–24 % received the welfare services they needed [5, 6].

The rights of PWDs have been increasingly recognised in Ghanaian Society, largely through advocacy spearheaded by PWDs themselves. A number of such persons have become successful role models in the society and the common perception that a person with a major form of disability must necessarily become a beggar by the roadside or otherwise live on the charity of others, is slowly being eroded. The passage of the “Person with Disability Act, 2006” in Ghana has further strengthen the rights of PWDs including the right to live in specialised establishments, participate in social, political, creative or recreational activities and access employment opportunities [7]. The law has also made provisions for punitive measures against persons who discriminate against PWDs or fail to provide conducive environment for their work. Full implementation of the law however remains a challenge because of limited financial resources, lack of commitment by political leaders and deeply entrenched negative socio-cultural beliefs and behaviours.

Although data on PWDs is limited in Ghana, the WHO, World Bank and the World Report on Disability estimates about 7–12 % population prevalence of disability with about 10.6 % being women and 6.2 % men [2, 8]. One study reported visual, auditory and physical impairments as the most common types of disabilities in Ghana whereas others identified neurocognitive disorders as being common in school children [9, 10]. Physical disabilities appear to be the most common among all forms of disabilities [11]. Most studies did not examine other forms of disabilities including mental and hearing impairments.

PWDs, like any other member of society, may sometimes find themselves in trouble with the law and end up in prison. The management of such prisoners in terms of provision of social amenities, access to family relations, access to recreational activities and many other opportunities has received little attention. Such individuals are therefore vulnerable to deficiencies in services such as health care, rehabilitation, social support and assistance; a situation which is contrary to the provisions of the Declaration on the Rights of the PWDs.

A survey of 1435 prisoners in the UK showed that 36 % of prisoners had disabilities of which 16 % suffered from anxiety or depression and 11 % had physical disability [12]. Similarly in the US, about 3 in 10 states and federal prisoners and 4 in 10 local jail inmates reported having at least one disability consisting of hearing, vision, cognitive, ambulatory, self-care, and independent living [13].

The situation in Africa is quite alarming because of the general neglect in the protection of the rights of prisoners with disabilities. The Correctional Services Act in South Africa for instance makes no provision for the special treatment of disabled prisoners [14]. The situation in Ghana is not different. Although, the regulations on the treatment of prisoners are set out, there is very little on the protection of the rights of disabled prisoners and detainees [15, 16]. From arrest through every phase of the criminal justice system, PWDs encounter a system not designed to handle large numbers of persons with disabilities. Although there have been a considerable increase in the number of prisoners in Ghana including those of PWDs, there have not been a commensurate increase in the infrastructural development of the prisons due to lack of funding to alter existing structures and the general lack of information on required facilities to aid PWDs [17, 18]. This therefore has the potential of worsening the plight of such persons.

Prisoners with disabilities have not been the focus of much study to date in the West African Sub-region and many parts of the world. Data on the occurrence of prisoners with disabilities, the type of disabilities and availability of rehabilitation support or assistive devices are not well documented in Ghana. The purpose of this current study therefore was to describe the socio-demographic characteristics of prisoners with disability, identify the types and causes of disability and describe the daily living experiences of PWDs serving various sentences in Ghanaian prisons, as a case study, in order to provide timely, relevant data and recommendations drawn from research for dealing with similar problems in the country and the sub-region.

## Methods

### Study area and population

This study was a descriptive cross-sectional survey conducted in the Male and Female Regional Prisons in Kumasi, Sunyani and the Nsawam Medium Security Prison, from November to December 2011. The Kumasi Central Prison is located in Kumasi; the capital of the Ashanti region of Ghana. Kumasi lies in the central forest belt of Ghana, situated at 6.72° N 1.6° W, approximately 290 m above sea level with a population of about 2,035,064 [19]. The Kumasi Central Prison was established in 1901 by the British colonial authorities to accommodate about 800 prisoners. The facility now houses around 1981 inmates [18]. The Sunyani Prisons is located in Sunyani; the capital of the Brong Ahafo region of Ghana and has a population of over 200,000 [19]. The prison was similarly established to accommodate 500 prisoners but currently accommodates about over 800 prisoners [20]. The Nsawam Prisons is located in Nsawam; a town in the Eastern Region of Ghana. The prison currently accommodates over 3000 inmates instead of its original capacity of 717 [18, 21].

### Sampling and recruitment methods

The prisons were selected based on their anticipated yield and proximity to the Centre for Disability and Rehabilitation Studies, (CEDRES), KNUST in Kumasi, Ashanti. CEDRES is the hub for disability studies and research established to train undergraduate and graduate students for disability practice in Ghana.

For purposes of the study, we defined disability as a term for impairments, activity limitations, and participation restrictions [1]. Assessment for impairments included physical, hearing, visual, speech, mental and hearing impairments. Questionnaires were constructed to centre on the key questions raised by the study. Variables such as Socio-Demographic Characteristics, Types and Causes of Disability, Daily Living Experiences, Availability and Accessibility of Recreational Facilities and Opportunities to Upgrade Knowledge and to Learn a vocation were included in the questionnaires.

The initial selection of PWDs was done by the prison officers based on the existing medical records available in the prisons on inmates. The records are screened by the prison officers and subjects identified as disable were selected. The selected prisoners were assembled in a designated area for the study. The purpose of the study was explained and a quick medical assessment was conducted by the principal investigator to ascertain the presence of a disability as presented by the prison officials before recruitment. All PWDs present consented to participate and were therefore recruited into the study.

### Ethical approval

Ethical approval was sought firstly from security officers by writing to the prison authority. The research team was then granted access to the prisons on set occasions by the Director-General of the Prison Service with strict prison security codes which was adhered to at all times of the study. Additional ethical approval was sought from the Committee of Human Research Publications and Ethics (CHRPE) of the School of Medical Sciences, Kwame Nkrumah University of Science and Technology (KNUST) /Komfo Anokye Teaching Hospital, through the Department of Community Health. The study took serious views of confidentiality, being very mindful of sensitivities to questions regarding disabilities, crime, jail sentences and security of data.

### Data management and statistical analysis

The study data were collected using structured questionnaires administered to the participants in English and local Ghanaian languages through face-to-face interviews. Data were captured using Microsoft Access Database System Software and secured using a password-protected computerized system due to the sensitivity of the data. Daily data entry and cleaning was done and backed-up on external drive which was kept under lock and key by the PI. The identity of PWDs was kept strictly anonymous. The analysis of the data was done using statistical package such as Microsoft excel 2010 for its descriptive statistics.]

## Results

### Socio-demographic characteristics

We screened 6114 records of prisoners of which 1852 (30.3 %) were from the Kumasi Central Prisons, 3483 (57 %) from the Nsawam Medium Security and 779 (12.8 %) from the Sunyani Central Prisons. A total of 99 PWDs were identified made up of 50 (50.5 %) from the Kumasi Central Prisons followed by 37 (37.4 %) from the Nsawam Medium Security Prisons and then 12 (12.1 %) from the Sunyani Central Prisons. Of the 99 PWDs, only 2 (2 %) were females whereas 97 (98 %) were males (Table 1).

**Table 1:** Characteristics of PWDs

Variable	Frequency	Percentage
Age (years)	(*N* = 99)	(%)
< 20	2	2.0
20–39	40	40.4
40–59	43	43.4
> 60	9	9.1
Missing age	5	5.1
Gender		0.0
Males	97	98.0
Females	2	2.0
Education level		0.0
No education	34	34.3
Middle school/JHS	52	52.5
Secondary school	6	6.1
Tertiary school	7	7.1
Occupation		0.0
Craftsman	15	15.2
Trader	12	12.1
Agriculture	20	20.2
Driver	10	10.1
Government employee	2	2.0
Unemployed	10	10.1
Other	30	30.3

The age distribution of the PWDs ranged from 18 to 79 years with the majority, 43 (43.4 %) between the ages of 40 to 59 years, followed by ages between 20 and 39 (40.4 %) while ages above 60 years (9.1 %) formed the minority. In terms of education, middle school/JSS leavers were in the majority, 52 (52.5 %) followed by those without any formal education 34 (34.3 %). Seven (7.1 %) had completed tertiary education with secondary school leavers 6 (6.1 %) in the minority.

### Types and causes of disability

Six types of disabilities were identified including physical, visual, hearing impairment, speech impairment, mental abnormality and albinism. The commonest type of disability was physical disability (81.8 %) followed by visual, hearing, speech, mental and albinism in descending order as depicted (Table 2). All forms of physical disabilities identified were mainly related to mobility. Majority of the disabilities were acquired (95 %) compared to a few that were congenital. Most of the acquired disabilities were as a result of trauma (68.8 %), infection (16.7 %), and drug related mental disabilities (6.3 %).

**Table 2:** Characteristics and types of disabilities

Disability	Frequency	Percentages
Types	*N* = 99	
Physical	81	81.8
Visual	7	7.1
Hearing	2	2.0
Speech	1	1.0
Mental	7	7.1
Albinism	1	1.0
Causes		
Trauma	68	68.7
Infection	17	17.2
Drugs	6	6.1
Stroke	3	3.0
Congenital	5	5.1
Required assistive devices		
Crutches	37	37.4
Wheel chair	29	29.3
Prosthetic limb	8	8.1
Others	4	4.0

Fifty (50.5 %) PWDs admitted they required an assistive device. The devices required were; crutches (37.5 %), wheel chairs (29.2 %), spectacles (20.8 %), prosthetic limbs (8.3 %) while 4.2 % indicated they required other different services. Further assessment showed that medications were only made available to 73 % of PWDs. Although PWDs were given access to recreational facilities, only 45 % accessed this. The recreational facilities were outdoor or indoors. The rest either did not make any effort to visit the facilities or were not in the known.

**Fig. 1:**
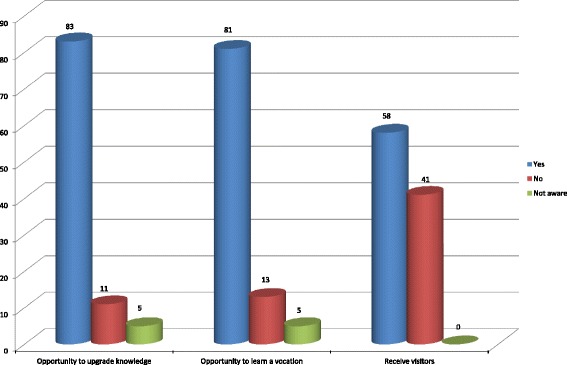
Distribution of opportunities to upgrade knowledge, to learn vocation and receive visitors

Our study also reported the availability of opportunities for prisoners to upgrade knowledge, learn vocation and receive visitors (Fig. 1). Majority of the study subjects; eighty three (83, 83.8 %) and 81 (81.8 %) respectively, admitted that they had the opportunity to upgrade knowledge and learn a vocation.

## Discussion

Knowledge of the prevalence of disabilities among prison inmates in developing countries especially Africa is heavily under-reported. The current study identified 1.6 % of prison inmates as suffering from various forms of disabilities with the commonest being physical disability The overall prevalence although generally low as compared to European [12] and WHO disability reports [2], the proportion is still significant and quite unique and this shows the extent of suffering prison inmates could be going through. The generally low prevalence in our study was because of our inability to capture the various forms of mental disabilities including anxiety, depression and psychotic disorders which is reported to contribute to 18 % of disabilities in the western world [12, 22, 23]. This limitation resulted from our reliance on the prisons medical records which probably under-reported specific cases of anxiety, depression and psychotic disorders. The design of our study in only recruiting subjects presented to us by prisons medical officers could also have contributed introduced selection bias leading to the low prevalence. It’s possible we might have missed some forms of disabilities including physical, mental etc. We however adopted this form of study design because of the highly controlled environment in the prison services and the perceived stigmatisation among disabled prisoners thus making it quite difficult to have access to all the prisoners. It’s possible the prevalence could have been higher if the current study had captured cases of disabilities. Although the overall prevalence is low, the major form of disability identified being physical has similarly been reported in other parts of Ghana [11].

Interviews with the disabled subjects revealed the causes of the physical disabilities as mostly due to infections and traumatic injuries suffered by prisoners possibly because of public abuses from robbery and theft. This scenario points to the need for government and other stakeholder organisations to promptly educate the public on the unwarranted abuses meted out to suspected criminals.

About half of the PWDs admitted they required assistive devices to aid them through their daily activities in the prison. Of these, most of the devices required were to assist in mobility. The need for these devices has become quite important because of the unfriendly nature of prison buildings to physically disabled subjects. Although confronted by huge economic challenges and budget deficits, it is also important that developing countries re-direct their focus towards improving the lives of prison inmates with physical challenges.

Our results also reported that many physically challenged prisoners with chronic ailments continually received drug supplies from medical personnel and in addition had access to recreational facilities and vocational education. Although these efforts deserve much commendation, more effort needs to be put in by government. The architectural design of the prisons does not generally favour PWDs as they have to compete with other prisoners for access to health care and recreational facilities. A recent report presented by the United Nations Rapporteur on Torture identified the lack of capacity of Kumasi and Nsawam prisons to deal with mental illness because of the general shortage of mental health services in the country. The report also pointed out situations of overcrowding with the two prisons holding 2.5–5 times its official capacity thus possibly depriving PWDs from accessing health and recreational or vocational activities [18]. Some other countries have reported inhumane conditions suffered by prisoners with disabilities thus denying them of basic rights to appropriate facilties, education, rehabilitation programmes and health care [24]. There is a need for the prisons to be improved so PWDs could gain increased access to rehabilitation programmes and othe social amenities.

About 41.4 % of prisoners indicated they had not received visitors over the duration of the study. The high percentage generally reflects the poor attitudinal behaviours that individuals including family relations demonstrate towards individuals with disabilities. A review by Tuakli-Wusornu described challenges with full socio-economic integration of persons with disabilities at the level of individual, family and the general society possibly due to social stigmatisation and socio-cultural beliefs of bad luck associated with such individuals [25]. Although this could generally apply to all prisoners, PWDs are likely to experience social exclusion because of the poor societal perceptions towards them especially when these disabilities resulted from criminal activities.

## Conclusion

The present study has demonstrated the occurrence and the forms of disabilities that prisoners with disabilities experience. Ghana and many other developing countries need to examine this in order to improve the livelihood of prisoners with disabilities. One major limitation of our study is our reliance on the prisons records for the selection of PWDs. The records seem inadequate as it did not completely detail or capture many forms of disability especially relating to mental disorders and other physical forms. The absence of a control group to adequately compare the effect of prison incarceration on the livelihood of PWDs could also not be addressed. We suggest further studies to explore other prisons, detailing the forms of disabilities and their conditions in other parts of Africa in order to make sound recommendations to stakeholders on behalf of PWDs. The use of a cross-sectional design incorporating all prisoners could also provide valuable information.

## Abbreviations

CHRPE, Committee of Human Research Publications and Ethics; KNUST, Kwame Nkrumah University of Science and Technology; PWDs, persons with disability
